# BioJava: an open-source framework for bioinformatics in 2012

**DOI:** 10.1093/bioinformatics/bts494

**Published:** 2012-08-09

**Authors:** Andreas Prlić, Andrew Yates, Spencer E. Bliven, Peter W. Rose, Julius Jacobsen, Peter V. Troshin, Mark Chapman, Jianjiong Gao, Chuan Hock Koh, Sylvain Foisy, Richard Holland, Gediminas Rimša, Michael L. Heuer, H. Brandstätter–Müller, Philip E. Bourne, Scooter Willis

**Affiliations:** ^1^San Diego Supercomputer Center, University of California San Diego, La Jolla, CA 92093, USA, ^2^European Bioinformatics Institute, Wellcome Trust Genome Campus, Hinxton, Cambridge CB10 1SD, UK, ^3^Bioinformatics Program, University of California San Diego, La Jolla, CA 92093, USA, ^4^College of Life Sciences, University of Dundee, Dundee DD1 5EH, UK, ^5^Department of Computer Science, University of Wisconsin-Madison, WI 53706, USA, ^6^Computational Biology Center, Memorial Sloan-Kettering Cancer Center, New York, NY 10065, USA, ^7^NUS Graduate School for Integrative Sciences and Engineering, Singapore 117597, Singapore, ^8^Genetics and Genomics Medicine of Inflammation, Montreal Heart Institute, Montreal, Quebec, Canada H1T 1C8, ^9^Eagle Genomics Ltd, Babraham Research Campus, Cambridge CB22 3AT, UK, ^10^Faculty of Mathematics and Informatics, Vilnius University, LT-03225 Vilnius, Lithuania, ^11^Harbinger Partners, Inc., St Paul, MN 55127, USA, ^12^University of Applied Sciences Upper Austria, 4232 Hagenberg, Austria, ^13^Skaggs School of Pharmacy and Pharmaceutical Sciences, University of California San Diego, La Jolla, CA 92093, USA and ^14^Genomics Core, Scripps Florida, Jupiter, FL 33458, USA

## Abstract

**Motivation:** BioJava is an open-source project for processing of biological data in the Java programming language. We have recently released a new version (3.0.5), which is a major update to the code base that greatly extends its functionality.

**Results:** BioJava now consists of several independent modules that provide state-of-the-art tools for protein structure comparison, pairwise and multiple sequence alignments, working with DNA and protein sequences, analysis of amino acid properties, detection of protein modifications and prediction of disordered regions in proteins as well as parsers for common file formats using a biologically meaningful data model.

**Availability:** BioJava is an open-source project distributed under the Lesser GPL (LGPL). BioJava can be downloaded from the BioJava website (http://www.biojava.org). BioJava requires Java 1.6 or higher. All inquiries should be directed to the BioJava mailing lists. Details are available at http://biojava.org/wiki/BioJava:MailingLists

**Contact:** andreas.prlic@gmail.com

## 1 INTRODUCTION

BioJava is an established open-source project driven by an active developer community (Holland *et al.*, 2008). It provides a framework for processing commonly used biological data and has seen contributions from >60 developers in the 12 years since its creation. The supported data range in scope from DNA and protein sequence information up to the level of 3D protein structures. BioJava provides various file parsers, data models and algorithms to facilitate working with the standard data formats and enables rapid application development and analysis.

The project is hosted by the Open Bioinformatics Foundation (OBF, http://www.open-bio.org), which provides the source code repository, bug tracking database and email mailing lists. It also supports projects SUCH AS BioPerl (Stajich *et al.*, 2002), BioPython (Cock *et al.*, 2009), BioRuby (Goto *et al.*, 2010), EMBOSS (Rice *et al.*, 2000) and others.

## 2 METHODS

Over the last 2 years, large parts of the original code base have been rewritten. BioJava 3 is a clear departure from the version 1 series. It now consists of several independent modules built using Maven (http://maven.apache.org). The original code has been moved into a separate biojava-legacy project, which is still available for backwards compatibility. In the following, we describe several of the new modules and highlight some of the new features that are included in the latest version of BioJava.

### 2.1 Core module

The core module provides classes to model nucleotide and amino acid sequences and their inherent relationships. Emphasis was placed on using Java classes and method names to describe sequences that would be familiar to the biologist and provide a concrete representation of the steps in going from a gene sequence to a protein sequence to the computer scientist.

BioJava 3 leverages recent innovations in Java. A sequence is defined as a generic interface, allowing the framework to build a collection of utilities which can be applied to any sequence such as multiple ways of storing data. In order to improve the framework’s usability to biologists, we also define specific classes for common types of sequences, such as DNA and proteins. One area that highlights this work is the translation engine, which allows the interconversion of DNA, RNA and amino acid sequences. The engine can handle details such as choosing the codon table, converting start codons to a methionine, trimming stop codons, specifying the reading frame and handling ambiguous sequences (‘R’ for purines, for example). Alternatively, the user can manually override defaults for any of these.

The storage of sequences is designed to minimize memory usage for large collections using a ‘proxy’ storage concept. Various proxy implementations are provided which can store sequences in memory, fetch sequences on demand from a web service such as UniProt or read sequences from a FASTA file as needed. The latter two approaches save memory by not loading sequence data until it is referenced in the application. This concept can be extended to handle very large genomic datasets, such as NCBI GenBank or a proprietary database.

### 2.2 Protein structure modules

The protein structure modules provide tools for representing and manipulating 3D biomolecular structures, with the particular focus on protein structure comparison. It contains Java ports of the FATCAT algorithm (Ye and Godzik, 2003) for flexible and rigid body alignment, a version of the standard Combinatorial Extension (CE) algorithm (Shindyalov and Bourne, 1998) as well as a new version of CE that can detect circular permutations in proteins (Bliven and Prlić, 2012). These algorithms are used to provide the RCSB Protein Data Bank (PDB) (Rose *et al.*, 2011) Protein Comparison Tool as well as systematic comparisons of all proteins in the PDB on a weekly basis (Prlić *et al.*, 2010).

Parsers for PDB and mmCIF file formats (Bernstein *et al.*, 1977; Fitzgerald *et al.*, 2006) allow the loading of structure data into a reusable data model. Notably, this feature is used by the SIFTS project to map between UniProt sequences and PDB structures (Velankar *et al.*, 2005). Information from the RCSB PDB can be dynamically fetched without the need to manually download data. For visualization, an interface to the 3D viewer Jmol (Hanson, 2010) http://www.jmol.org/ is provided. Work is underway for better interaction with the RCSB PDB viewers (Moreland *et al.*, 2005).

### 2.3 Genome and sequencing modules

The genome module is focused on the creation of gene sequence objects from the core module by supporting the parsing of GTF files generated by GeneMark (Besemer and Borodovsky, 2005), GFF2 files generated by GeneID (Blanco and Abril, 2009) and GFF3 files generated by Glimmer (Kelley *et al.*, 2011). The gene sequences can then be written out as a GFF3 format for importing into GMOD (Stein *et al.*, 2002). A separate sequencing module provides memory efficient, low level and streaming I/O support for several common variants of the FASTQ file format from next generation sequencers (Cock *et al.*, 2010).

### 2.4 Alignment module

The alignment module supplies standard algorithms for sequence alignment and establishes a foundation to perform progressive multiple sequence alignments. For pairwise alignments, an implementation of the Needleman–Wunsch algorithm computes the optimal global alignment (Needleman and Wunsch, 1970) and the Smith–Waterman algorithm calculates local alignments (Smith and Waterman, 1981). In addition to these standard pairwise algorithms, the module includes the Guan–Uberbacher algorithm to perform global sequence alignment efficiently using only linear memory (Guan and Uberbacher, 1996). This routine also allows predefined anchors to be manually specified that will be included in the alignment produced. Any of the pairwise routines can also be used to perform progressive multiple sequence alignment. Both pairwise and multiple sequence alignments output to standard alignment formats for further processing or visualization.

### 2.5 ModFinder module

**Fig. 1.**
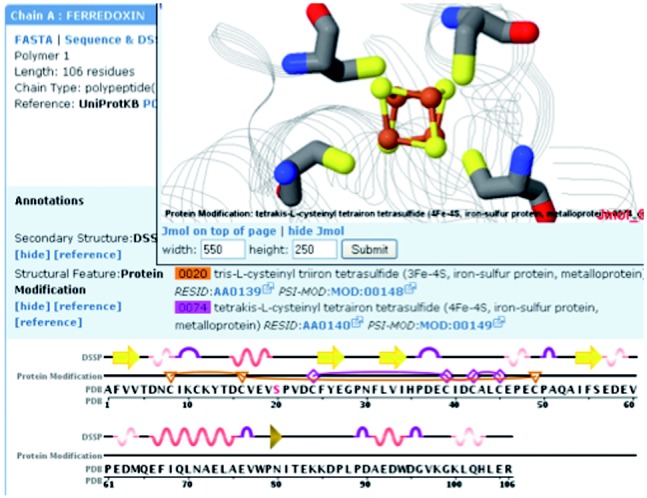
An example application using the ModFinder module and the protein structure module. Protein modifications are mapped onto the sequence and structure of ferredoxin I (PDB ID 1GAO; Chen *et al.*, 2002). Two possible iron–sulfur clusters are shown on the protein sequence (3Fe–4S (F3S): orange triangles/lines; 4Fe–4S (SF4): purple diamonds/lines). The 4Fe–4S cluster is displayed in the Jmol structure window above the sequence display

The ModFinder module provides new methods to identify and classify protein modifications in protein 3D structures. More than 400 different types of protein modifications (phosphorylation, glycosylation, disulfide bonds metal chelation, etc.) were collected and curated based on annotations in PSI-MOD (Montecchi-Palazzi *et al.*, 2008), RESID (Garavelli, 2004) and RCSB PDB (Berman *et al.*, 2000). The module provides an API for detecting protein modifications within protein structures. Figure 1 shows a web-based interface for displaying modifications which was created using the ModFinder module. Future developments are planned to include additional protein modifications by integrating other resources such as UniProt (Farriol-Mathis *et al.*, 2004).

### 2.6 Amino acid properties module

The goal of the amino acid properties module is to provide a range of accurate physicochemical properties for proteins. The following peptide properties can currently be calculated: molecular weight, extinction coefficient, instability index, aliphatic index, grand average of hydropathy, isoelectric point and amino acid composition.

To aid proteomic studies, the module includes precise molecular weights for common isotopically labeled or post-translationally modified amino acids. Additional types of PTMs can be defined using simple XML configuration files. This flexibility is especially valuable in situations where the exact mass of the peptide is important, such as mass spectrometry experiments.

### 2.7 Protein disorder module

BioJava now includes a port of the Regional Order Neural Network (RONN) predictor (Yang *et al.*, 2005) for predicting disordered regions of proteins. BioJava’s implementation supports multiple threads, making it ∼3.2-times faster than the original C implementation on a modern quad-core machine.

The protein disorder module is distributed both as part of the BioJava library and as a standalone command line executable. The executable is optimized for use in automated analysis pipelines to predict disorder in multiple proteins. It can produce output optimized for either human readers or machine parsing.

### 2.8 Web service access module

More and more bioinformatics tools are becoming accessible through the web. As such, BioJava now contains a web services module that allows bioinformatics services to be accessed using REST protocols. Currently, two services are implemented: NCBI Blast through the Blast URLAPI (previously known as QBlast) and the HMMER web service at hmmer.janelia.org (Finn *et al.*, 2011).

## 3 CONCLUSION

The BioJava 3 library provides a powerful API for analyzing DNA, RNA and proteins. It contains state-of-the-art algorithms to perform various calculations and provides a flexible framework for rapid application development in bioinformatics. The library also provides lightweight interfaces to other projects that specialize in visualization tools. The transition to Maven made managing external dependencies much easier, allowing the use of external libraries without overly complicating the installation procedure for users.

The BioJava project site provides an online cookbook which demonstrates the use of all modules through short recipes of common tasks. We are looking forward to extending the BioJava 3 library with more functionality over the coming years and welcome contributions of novel components by the community.
